# Clinical and Microbiological Characteristics of Deep Neck Abscesses in Pediatrics: Analysis of a Case Series from a 3rd Level Pediatric Hospital

**DOI:** 10.3390/children10091506

**Published:** 2023-09-04

**Authors:** Marcello Mariani, Carolina Saffioti, Alessio Mesini, Candida Palmero, Roberto D’Agostino, Sabrina Garofolo, Andrea Rossi, Maria Beatrice Damasio, Elio Castagnola

**Affiliations:** 1 Infectious Diseases Unit, IRCCS Istituto Giannina Gaslini, 16147 Genoa, Italy; 2 Microbiology Laboratory, IRCCS Istituto Giannina Gaslini, 16147 Genoa, Italy; 3 Otolaryngology Unit, IRCCS Istituto Giannina Gaslini, 16147 Genoa, Italy; 4 Neuroradiology Unit, IRCCS Istituto Giannina Gaslini, 16147 Genoa, Italy; 5 Radiology Unit, IRCCS Istituto Giannina Gaslini, 16147 Genoa, Italy

**Keywords:** abscess, neck infections, NSDAIDs, peritonsillar, retropharyngeal, parapharyngeal

## Abstract

As there is currently no consensus on managing deep neck infections in pediatric populations, we report a case series from a large pediatric hospital. Clinical data of patients discharged from Istituto Gaslini-Children’s Hospital from January 2014 to June 2020 with peritonsillar, parapharyngeal, or retropharyngeal abscess diagnoses were collected. A total of 59 patients were identified. Patients underwent surgical drainage in 47% of cases. Streptococcus mitis/oralis was the most isolated pathogen. Surgically treated patients did have larger abscesses compared to others, but there were no differences in the duration of hospitalization. Children who received NSAIDs at home had significant delays in diagnosis (median 4 vs. 1.5 days, *p* = 0.008). In our experience, clinical presentation of DNIs is often evocative, but evaluation should include imaging with CT/MRI. Surgery is effective in larger abscesses, allowing for etiological diagnosis with consequent antibiotic adjusting. From an anamnestic point of view, home medications such as NSAIDs could delay diagnosis.

## 1. Introduction

Deep neck infections (DNIs) are rare but life-threatening infections localized among neck loggias and fascial planes. DNIs in childhood are more frequent than in adults due to the higher frequency of upper respiratory tract infections, which represent important contributing factors [1,2], and the presence of lymph nodes in the retropharyngeal space, which undergo regression after childhood [3]. From an anatomical point of view, at least 11 deep neck spaces created by the complex web of fascial planes can be recognized: the adhesion of these bands to hyoid bone contributes to the creation of the most important obstacle to the spread of inflammatory processes [3]. Deep neck spaces are then classified into three groups according to their relative position to the hyoid bone: suprahyoid (peritonsillar, submandibular, parapharyngeal, temporal, buccal, and parotid spaces), with full-length extension in the neck (retropharyngeal, prevertebral, and carotid spaces), anterior (or pretracheal) space, or infrahyoid area (below the hyoid bone). The peritonsillar space is the most frequently affected in children [4]. Other common DNI localizations are para- and retropharyngeal loggias, where suppurative processes may not find obstacles to their extension, resulting in potentially fatal conditions such as airway compression, jugular septic thrombosis (Lemierre’s syndrome), and mediastinitis [5].

Oral streptococci such as *Streptococcus mitis*/*oralis* and *Streptococcus pyogenes* are the most frequently isolated pathogens, but it is not uncommon to find anaerobes as *Prevotella* spp. [2,6]. The clinical presentation of DNIs in children is frequently characterized by pharyngodynia, stiff neck, odynophagia, and variable solid and/or liquid dysphagia [7]. Trismus, sialorrhea, and voice changes may also be observed upon physical examination. Diagnosis is usually clinical, and can be corroborated by computed tomography (CT) or magnetic resonance imaging (MRI) [4,7]. As already underlined by a previous large Italian study [6], there is currently no consensus on the management of DNIs in pediatric cases. The cornerstones of medical therapy are represented by analgesics and antibiotics [8]. Among the latter, those effective both on streptococci and anaerobic microorganisms, such as penicillins or the combination of ceftriaxone/cefotaxime and metronidazole, should be considered. Steroids have been proposed for anti-edema and anti-inflammatory purposes in association with antibiotics, but their role is still controversial [9]. On the other hand, surgical management involves incision and drainage techniques or needle aspiration [10]. Drainage material can be cultured in order to adapt antibiotic therapy on isolates and antibiograms [11]. Although the advancement of medical techniques has led to a reduction in life-threatening complications [12], the management of pediatric DNIs is still based on local recommendations.

The aim of our study is to retrospectively evaluate a large cohort of pediatric DNIs to evaluate the population, clinical, and microbiological characteristics.

## 2. Materials and Methods

Clinical records of patients discharged from to Istituto Giannina Gaslini Children’s Hospital, Genoa, Italy, from January 2014 to June 2020, each with a diagnosis of “peritonsillar abscess”, “parapharyngeal abscess”, or “retropharyngeal abscess” and an absence of immune deficiencies, were considered to be eligible. For each patient, the following demographic and clinical data were recorded: sex, year of birth, hospitalization date, days between symptom onset and hospital admission (diagnosis delay), hospitalization length, and clinical presentation (presence of trismus/dysphagia upon physical examination or fever > 38 °C). Laboratory data: C reactive protein (CRP) and leukocyte count. Treatment was administered before hospitalization (non-steroidal anti-inflammatory drugs (NSAIDs) or antibiotics). Management data: magnetic resonance imaging (MRI) or computed tomography (CT), performed during hospitalization (if any). In the case of abscess detection, the volume was estimated by means of the ellipsoid formula (4/3 π R1 × R2 × R3) [13]. Therapy: only medical or medical plus surgical, and pathogens that were isolated from drainage cultures. Descriptive statistics were carried out for population, clinical, and microbiological data. Mean and 95% confidence intervals (95% CI) were used for normally distributed continuous variables (identified by Shapiro–Wilk’s test), while the median and interquartile range (IQR) were used for non-normally distributed data. Absolute numbers and percentages were used for the categorical variables. To compare groups, parametric (*t*-test) or non-parametric (Mann–Whitney or Kruskal–Wallis) tests were used according to the distribution variables. Bonferroni’s rule was applied in cases of repeated analyses of the same data. All statistical tests were carried out using spreadsheets (Excel ver. 2307, Office 365, Microsoft Corp., Redmond, WA, USA) and Jamovi statistical software ver. 2.3.28 “solid” (www.jamovi.org, last access 10 August 2023).

## 3. Results

**Figure 1:**
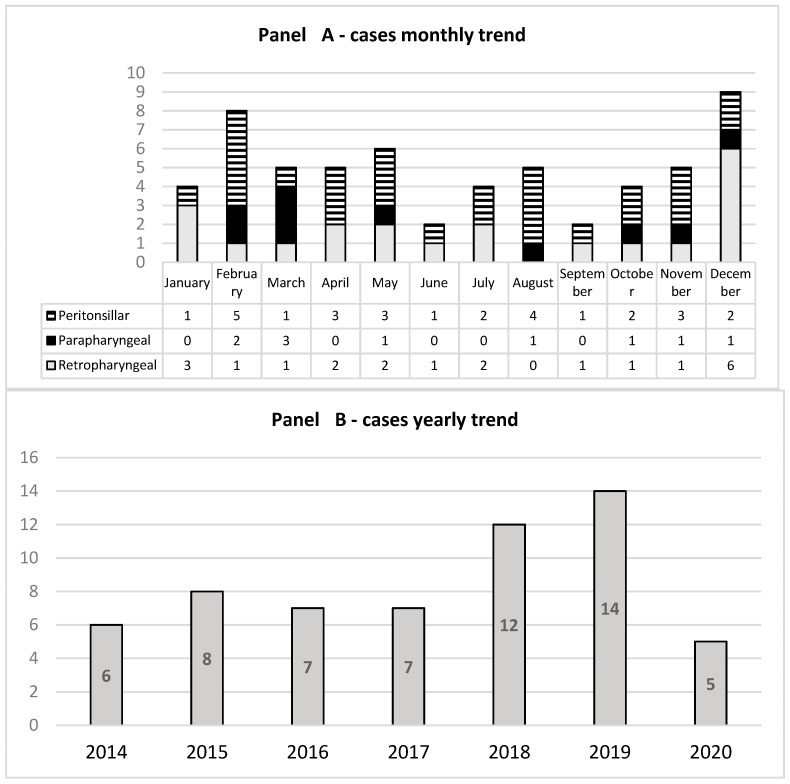
Number of hospitalizations per month (**panel** (**A**)) and per year (**panel** (**B**)).

During the study period, a total of 59 patients were identified, including 23 females (39%). DNIs were 10/59 (16.9%) parapharyngeal, 28/59 (47.5%) peritonsillar, and 21/59 (35.6%) retropharyngeal abscesses, respectively. The median age at diagnosis for parapharyngeal abscesses was 6 years (IQR 5; 9), 10 years for peritonsillar (IQR 7; 14), and 3 for retropharyngeal (IQR 2; 10). The age difference between the peritonsillar and retropharyngeal abscess groups was statistically significant (*p* = 0.01). A total of 31 (52.5%) cases were observed from November to March; moreover, there was an increasing trend in the number of DNIs in 2018 and 2019 (Figure 1).

### 3.1. Clinical and Laboratory Features

In 49/59 cases (83.1%), the patient was admitted to the emergency room for fever > 38 °C. Trismus, sialorrhea, and swallowing difficulty were highlighted in 44/59 patients (74.6%); in 7/59 (11.9%) cases, no otolaryngological symptoms were reported, while in 8/59 (13.6%), the data were unknown. There were no differences in clinical presentation between DNI sites. Table 1 reports the patients’ demographic, clinical, and laboratory data, showing a mean CRP of 13.25 mg/dL in parapharyngeal, 6.85 mg/dL in peritonsillar, and 12.8 mg/dL in retropharyngeal abscesses (normal value < 0.46 mg/dL). The mean CRP difference between peritonsillar and retropharyngeal abscesses was statistically significant (*p* = 0.05 corrected for Bonferroni test). The blood count showed a median leukocyte count of 20,000/mmc (IQR 14,000; 22,000) (normal value 4000–10,000/mmc), with neutrophilia at 16,000/mmc (IQR 11,000; 18,000) (normal value 2000–6000/mmc). No statistically significant differences were found in leucocyte counts between different abscess locations.

### 3.2. Microbiological Features

*S. pyogenes* was detected by means of rapid antigen in 50% of the tested cases (9/18), while in no cases was *S. aureus* detected by means of pharyngeal swab. Nasopharyngeal multiplex real-time PCR swabs for bacteria were performed in 22/59 patients (37%). Of these, 2/22 (9%) resulted positive for *S. pneumoniae* and 1/22 (4.5%) for *Haemophilus influenzae*. These pathogens, however, were not found in surgical drainage cultures.

Patients underwent surgical drainage in 28/59 cases (47%), and 30 pathogens were isolated (Table 2).

*Viridans* streptococci (n = 19, 63%) were the most frequent, and among them, *Streptococcus mitis*/*oralis* was the most represented (n = 8, 27%). Methicillin-susceptible *Staphylococcus aureus* and *Streptococcus pyogenes* were identified in five cases each. The microbiological data are summarized in Table 2.

### 3.3. Management

MRI or CT was performed in 53/59 patients (89.8%), and demonstrated the presence of abscess in 47/53 cases (90.4%). Antibacterial therapy was administered to all patients, but in 28/47 (59.6%), surgery was necessary as well. Noteworthy, patients who needed surgery had significantly larger abscesses detected at imaging (median volume 72.073 cm^3^, IQR 21.308–114.924, vs. median 21.771 cm^3^, IQR 5.927–27.171, *p* = 0.006). On the contrary, patients who underwent surgery had a shorter, but not statistically significant, duration of hospitalization (median: 7 days; IQR: 5–13 vs. median: 9 days; IQR: 7–14, *p* = 0.114).

With regard to the treatment received before hospitalization, oral amoxicillin/clavulanate was administered in 19/59 cases (32.2%), clarithromycin in 2/59 (3.4%), and cephalosporins in 3/59 (5.1%). Domestic self-medication with an antipyretic drug was reported in 30 cases: 17/30 (56.7%) received at least one dose of ibuprofen (NSAIDS). Noteworthily, children who received NSAIDS at home had a statistically significant diagnosis delay (median 4 days, IQR 3–5; vs. 1.5 days, IQR1–4; *p* = 0.008). No patients received oral steroids at home.

## 4. Discussion

In this retrospective, 6.5-year-long study, we confirmed that DNIs as a relatively rare complication in pediatrics, considering the extreme frequency of respiratory tract infections in this population [14]. From an epidemiological point of view, despite the absence of a strict seasonal distribution, about 50% of our patients were admitted between November and March, the time of the year with higher incidence of influenza-like-illnesses. Furthermore, considering the annual prevalence of invasive *S. pyogenes* infections, as indicated by other international studies [15], a bimodal peak in winter and late spring could also fit with our series. Patients with peritonsillar abscesses were older than those hospitalized for retropharyngeal (10 years vs. 3), probably due to the presence of retro- and parapharyngeal lymph node chains, which undergo spontaneous regression in later ages [16]. The typical presentation was represented by fever (83% of cases) associated with trismus or dysphagia (74.6%). No characteristics of signs or symptoms for the different abscess forms were identified. Aside from physical examination, imaging was an important diagnostic instrument capable of identifying abscess presence in more than 90% of patients. Furthermore, in retropharyngeal abscesses, CRP was higher (12.8 mg/dL vs. 6.85 mg/dL), emphasizing the systemic relevance of retropharyngeal abscesses. Microbiological diagnosis was difficult, but, when identified, the etiological agents were usually associated with DNIs. An important role could be played by the streptococcal rapid antigen test, which, due to its high sensitivity and specificity [17], could address issues with diagnosis. In our case series, noninvasive bacterial culture/PCR throat swab showed extremely limited usefulness: a very small number of pathogens were identified with this method, and in no case were these confirmed on drainage material culture. Noteworthy, differently from other reports [18], MRSA was not identified among the causes of DNIs in our series. The absence of anaerobes may be due to the difficulties in collecting culture samples in the correct way. Surgical drainage was performed in about 60% of cases and was decided by a case-by-case evaluation of abscess size and clinical course, but in any case, the larger abscesses were those most frequently undergoing surgical drainage, confirming previous data [19]. Surgery had an impact on hospitalization, with a tendency for a shorter, even if not statistically significant, duration in patients undergoing surgery. This contrasts with the results of a large study conducted in the United States, where patients treated surgically showed not only higher hospital costs, but also median lengths of stay one day longer than patients treated medically [20]. This difference could be due either to local epidemiology or to the fact that all patients in our cohort were also managed medically with e.v. antibiotics, reserving surgery only for selected cases. A final comment regarding the association between DNIs and NSAIDs administered at home is that the time from symptom onset and hospitalization was significantly longer in children treated with NSAIDs, who, in addition, presented a trend toward abscesses of greater dimensions at imaging. This observation has not yet been reported. Even if a definite causal relationship exists between home therapy with NSAIDs and an increased risk of DNIs, it is possible that a diagnostic delay could result from the pain-relieving and anti-inflammatory effect of these drugs, masking the symptoms and clinical signs (as dysphagia) of DNIs. The limitations of our study are the retrospective and single-center design, as well as the variability in clinical judgement among specialists; different patients, even if they are similar in presentation or in clinical course, could have been managed differently depending on the admission ward or other variables related to inpatient organization. A further limitation on the microbiological diagnosis side lies in the fact that it is technically difficult to culture anaerobic pathogens following neck surgery, as even a small amount of air in the aspirator could prevent the growth of anaerobic strains.

## 5. Conclusions

The management of DNIs should primarily consist of e.v. antibiotics active on common oral pathogens, without coverage for MRSA; among noninvasive microbiological tests, only rapid antigen detection for Group A Streptococcus could play a role. Imaging with CT or MRI is useful in identifying the presence of abscesses, estimating their size, and thus deciding whether to proceed with surgery. Patients exposed to NSAIDs could have delays in diagnosis and, consequently, have larger neck abscesses. As rapid management is often crucial in DNIs in order to avoid serious complications, early imaging and surgical approaches could be indicated for patients with a history of domiciliary therapy with NSAIDs.

## Figures and Tables

**Table 1:** Clinical presentation and population differences between DNIs in children.

		Parapharyngeal	Peritonsillar	Retropharyngeal	Total	*p*
**Age (years)**	Median	6.38	10.7	3.05		0.01
	1° Q	5.06	6.53	2		
	3° Q	8.92	13.7	9.69		
**Sex**	M	7	19	10	36	0.29
	F	3	9	11	23	
	Total	10	28	21	59	
**Clinical** **presentation**	Trismus	8	21	15	44	0.897
	No ENT symptoms	1	4	2	7	
	Total	9	25	17	51	
	Fever	8	25	16	49	0.463
	Apyrexia	2	3	5	10	
	Total	10	28	21	59	
**CRP (mg/d** **L** **)**	Mean	13.3	6.85	12.8		0.05
	IC 95%	8–18	3–10	9–16		
**WBC** **(/mmc)**	Median	21,000	17,000	18,000		0.160
	1st Q	20,000	14,000	16,000		
	3rd Q	26,000	21,000	26,000		

Q = quartile.

**Table 2:** Isolates from drainage cultures.

Drainage Culture Pathogen	Parapharyngeal	Peritonsillar	Retropharyngeal	Total
Streptococcus mitis	1	4	2	7
Methicillin-sensitive Staphylococcus aureus (MSSA)		5		5
Streptococcus pyogenes		5		5
Streptococcus salivarius	1	2	1	4
Streptococcu sanginosus		2		2
Streptococcus parasanguinis		1	1	2
Streptococcus vestibularis		2		2
Methicillin-sensitive Staphylococcus epidermidis (MSSE)			1	1
Streptococcus oralis		1		1
Streptococcus viridans		1		1
Negative culture			1	1

## Data Availability

Data are available upon request to authors.

## References

[1] Wald R., Wald E.R., Guerra N., Byers C. (1991). Upper Respiratory Tract Infections in Young Children: Duration of and Frequency of Complications. Pediatrics.

[2] Jain A., Singh I., Meher R., Raj A., Rajpurohit P., Prasad P. (2018). Deep Neck Space Abscesses in Children below 5 Years of Age and Their Complications. Int. J. Pediatr. Otorhinolaryngol..

[3] Vieira F., Allen S.M., Stocks R.M.S., Thompson J.W. (2008). Deep Neck Infection. Otolaryngol. Clin. N. Am..

[4] Baldassari C., Shah R.K. (2012). Pediatric Peritonsillar Abscess: An Overview. Infect. Disord. Drug Targets.

[5] Baldassari C.M., Howell R., Amorn M., Budacki R., Choi S., Pena M. (2011). Complications in Pediatric Deep Neck Space Abscesses. Otolaryngol.—Head Neck Surg..

[6] Donà D., Gastaldi A., Campagna M., Montagnani C., Galli L., Trapani S., Pierossi N., De Luca M., D’Argenio P., Tucci F.M. (2021). Deep Neck Abscesses in Children: An Italian Retrospective Study. Pediatr. Emerg. Care.

[7] Esposito S., De Guido C., Pappalardo M., Laudisio S., Meccariello G., Capoferri G., Rahman S., Vicini C., Principi N. (2022). Retropharyngeal, Parapharyngeal and Peritonsillar Abscesses. Children.

[8] Mayor G.P., Millán J.M.S., Martínez-Vidal A. (2001). Is Conservative Treatment of Deep Neck Space Infections Appropriate?. Head Neck.

[9] Tansey J.B., Hamblin J., Mamidala M., Thompson J., Mclevy J., Wood J., Sheyn A. (2020). Dexamethasone Use in the Treatment of Pediatric Deep Neck Space Infections. Ann. Otol. Rhinol. Laryngol..

[10] Kirse D.J., Roberson D.W. (2001). Surgical Management of Retropharyngeal Space Infections in Children. Laryngoscope.

[11] Pong A.L., Bradley J.S. (2005). Guidelines for the Selection of Antibacterial Therapy in Children. Pediatr. Clin. N. Am..

[12] Hoffmann C., Pierrot S., Contencin P., Morisseau-Durand M.P., Manach Y., Couloigner V. (2011). Retropharyngeal Infections in Children. Treatment Strategies and Outcomes. Int. J. Pediatr. Otorhinolaryngol..

[13] Betsch A., Wiskirchen J., Trübenbach J., Manncke K.H., Belka C., Claussen C.D., Duda S.H. (2002). CT-Guided Percutaneous Drainage of Intra-Abdominal Abscesses: APACHE III Score Stratification of 1-Year Results. Eur. Radiol..

[14] Jain N., Lodha R., Kabra S.K. (2001). Upper Respiratory Tract Infections. Indian J. Pediatr..

[15] Lamagni T.L., Darenberg J., Luca-Harari B., Siljander T., Efstratiou A., Henriques-Normark B., Vuopio-Varkila J., Bouvet A., Creti R., Ekelund K. (2008). Epidemiology of Severe Streptococcus Pyogenes Disease in Europe. J. Clin. Microbiol..

[16] Page C., Biet A., Zaatar R., Strunski V. (2008). Parapharyngeal Abscess: Diagnosis and Treatment. Eur. Arch. Oto-Rhino-Laryngol..

[17] Stewart E.H., Davis B., Clemans-Taylor B.L., Littenberg B., Estrada C.A., Centor R.M. (2014). Rapid Antigen Group A Streptococcus Test to Diagnose Pharyngitis: A Systematic Review and Meta-Analysis. PLoS ONE.

[18] Abdel-Haq N., Quezada M., Asmar B.I. (2012). Retropharyngeal Abscess in Children: The Rising Incidence of Methicillin-Resistant Staphylococcus Aureus. Pediatr. Infect. Dis. J..

[19] Wong D.K.C., Brown C., Mills N., Spielmann P., Neeff M. (2012). To Drain or Not to Drain—Management of Pediatric Deep Neck Abscesses: A Case-Control Study. Int. J. Pediatr. Otorhinolaryngol..

[20] Lipsett S.C., Porter J.J., Monuteaux M.C., Watters K., Hudgins J.D. (2021). Variation in the Management of Children With Deep Neck Infections. Hosp. Pediatr..

